# On the Hand-feeding of Infants

**Published:** 1875-02-01

**Authors:** Eustace Smith

**Affiliations:** London


### ON THE HAND-FEEDING OF INFANTS.

BY EUSTACE SMITH, M.D., LONDON.

THERE are few subjects of greater interest, or of which it is more important, in a sanitary sense, to possess an accurate knowledge than that which relates to the feeding and nurture of infants. Many mothers are unable to nurse their babies, and there is an increasing dislike to transfer maternal duties to a hireling; consequently, the question how best to provide a fitting diet for a being whose digestive powers are feeble and immature, but whose growth and healthy development are dependent upon a suitable supply of nourishment, is one to which it is of the utmost importance to furnish a correct answer.

The mortality among children under the age of twelve months is enormous; and of these deaths, a large proportion might be prevented by a wider diffusion of knowledge, of one of the least difficult of subjects. The rules for the efficient nourishment of infants, are plain and simple, and the application of them, although requiring tact and judgment, is yet not a matter which ought to occasion any extraordinary embarrassment.

The great principle at the bottom of all successful feeding—viz., that an infant is nourished in proportion to his power of digesting the food with which he is supplied, and not in proportion to the quantity of nutritive material which he may be induced to swallow—is so obviously true, that an apology might almost seem to be required for stating so self-evident a proposition; but experience shows that this simple truth is one which in practice is constantly lost sight of. That that child thrives best who is most largely fed, and that the more solid the food the greater its nutritive power, are two articles of faith so firmly settled in the minds of many persons, that it is very difficult indeed to persuade them to the contrary.

To them wasting in an infant, merely suggests a larger supply of more solid food; every cry means hunger, and must be quieted by an additional meal. To take a common case: A child, weakly perhaps to begin with, is filled with a quantity of solid food which he has no power of digesting. His stomach and bowels revolt against the burden imposed upon them, and endeavor to get rid of the offending matter by vomiting and diarrhœa; a gastro-intestinal catarrh is set up, which still further reduces the strength—every meal causes a return of the sickness; the bowels are filled with fermenting matter, which excites violent griping pains, so that the child rests neither night nor day; after a longer or shorter time, he sinks, worn out by pain and exhaustion, and is then said to have died from "consumption of the bowels."

Cases such as the above are but too common, and must be painfully familiar to every physician who has much experience of the diseases of children. When seen sufficiently early, the treatment of the derangement is simple and the improvement immediate, but it unfortunately often happens, especially among the poorer classes, that application for advice is delayed until the child's strength has been reduced to the lowest point, and all our efforts to remedy the mischief may in such cases prove unavailing.

The disastrous results of ignorant attempts to supply a substitute for human milk, have brought the whole practice of hand-feeding into disrepute; but if a food be judiciously selected, with a correct appreciation of infant wants, and an accurate estimate of infant powers of digestion, there is no reason why a child fed artificially, with judgment, should not thrive as well as one suckled naturally at his mother's breast. The food we select for the diet of an infant should be nutritious in itself, but it should also be given in a form in which the child is capable of digesting it, otherwise we may fill him with food without in any way contributing to his nutrition, and actually starve the body, while we load the stomach to repletion. No food can be considered suitable to the requirements of the infant, unless it not only possesses heat-giving and fat-producing properties, but also contains material to supply the waste of the nitrogenous tissues; therefore, a merely starchy substance, such as arrowroot, which enters so largely into the diet of children, especially among the poor, is a very undesirable food for infants, unless given in very small quantities, and mixed largely with milk.

The most perfect food for children, the only one, indeed, which can be trusted to supply in itself all the necessary elements of nutrition, in the most digestible form, is milk. In it are contained nitrogenous matter in the curd, fat in the cream, besides sugar, and the salts which are so essential to perfect nutrition. The milk of different animals varies to a certain extent in the proportion of the several constituents, some containing more curd, others more cream and sugar; but the milk of the cow, which is always readily obtainable, is the one to which recourse is usually had, and when properly prepared, this is perfectly efficient for the purpose required. Cow's milk contains a larger proportion of curd and cream, but less sugar, than is found in human milk, and these differences can be immediately remedied by dilution with water, and the addition of cane or milk sugar in sufficient quantity to supply the necessary sweetness. But there is another and more important difference between the two fluids which must not be lost sight of. If we take two children, the one fed on cow's milk and water, the other nursed at his mother's breast, and produce vomiting directly after a meal by friction over the abdomen, we notice a remarkable difference in the matters ejected. In the first case, we see the curd of the milk coagulated into a firm, dense lump; while in the second, the curd appears in the form of minute flocculent loosely connected granules. The demands made upon the digestive powers in these two cases is very different, and the experiment explains the difficulty often experienced by infants in digesting cow's milk, however diluted it may be, for the addition of water alone will not hinder the firm clotting of the curd. In order to make such milk perfectly satisfactory as a food for new-born infants, further preparation is required, and there are two ways in which the difficulty may be overcome.

The first method consists in adding an alkali, as lime-water, to the milk. To be of any service, however, the quantity added must be considerable, and one or two teaspoonfuls—the addition usually made to a bottleful of milk and water—is quite insufficient to effect the object desired. Lime-water contains only half a grain of lime to the fluid ounce; of this solution, so small a quantity as two teaspoonfuls would be scarcely sufficient even to neutralize the natural acidity of the milk. But it is necessary to do much more than this. Lime-water, no doubt, acts by partially neutralizing the gastric juice—the rennet naturally existing in the child's stomach—so that clotting of the curd is in great part prevented, and the milk passes little changed out of the stomach to be fully digested by the intestinal secretions in the bowels. To attain this object, at least a third part of the mixture should consist of lime-water. For a new-born infant, two tablespoonfuls of milk may be diluted with an equal quantity of plain filtered water, and then be alkalinized by two tablespoonfuls of lime-water. This mixture, of which only a third part is milk, can be sweetened by the addition of a teaspoonful of milk-sugar. If thought desirable, a teaspoonful of cream may be added. The whole is then put into a perfectly clean feeding-bottle, and is heated to a temperature of about 95° Fahr., by steeping the bottle in hot water; when warmed, it is ready for use. The proportion of milk can be gradually increased as the child gets older.

There is another plan by which the caseine of cow's milk may be rendered digestible; it is by adding to the milk a small quantity of some thickening substance, such as barley-water, isinglass, or even one of the ordinary farinaceous foods. The action of all of these is the same, and is an entirely mechanical one. The thickening substance separates the particles of curd, so that they cannot run together into a solid lump, but coagulate separately into a multitude of small masses. By this means the curd is made artificially to resemble the naturally light clot of human milk, and is almost as readily digested by the infant.

Although any thickening matter will have the mechanical effect desired of separating the particles of curd, yet it is not immaterial what substance is chosen. The question of the farinaceous feeding of infants is a very important one, for it is to an excess of this diet that so many of their derangements may often be attributed. Owing to a mistaken notion that such foods are peculiarly light and digestible—a notion so widely prevalent that the phrase "food for infants" has become almost synonymous with farinaceous matter—young babies are often fed as soon as they are born with large quantities of corn-flour or arrowroot, mixed sometimes with milk, but often with water alone. Now, starch, of which all the farinæ so largely consist, is digested principally by the saliva, aided by the secretion from the pancreas, which convert the starch into dextrine and grape-sugar previous to absorption. But the amount of saliva formed in the new-born infant is excessively scanty, and it is not until the fourth month that the secretion becomes fully established. Again, according to the experiments of Korowin, of St. Petersburg, the pancreatic juice is almost absent in a child of a month old; even in the second month, its secretion is very limited, and has little action upon starch. It is only at the end of the third month that its action upon starch becomes sufficiently powerful to furnish material for a quantitative estimation of the sugar formed. Therefore, before the age of three months, a farinaceous diet is not to be recommended—is even to be strongly deprecated, unless the starchy substance be given with great caution and in very small quantities. If administered recklessly, as it too often is, the food lies undigested in the bowels, ferments, and sets up a state of acid indigestion, which in so young and feeble a being, may lead to the most disastrous consequences. In fact, the deaths of many children under two or three months old, can often be attributed to no other cause than a purely functional abdominal derangement, excited and maintained by too liberal feeding with farinaceous foods. There is, however, one form of food, which, although farinaceous, is yet well digested even by young infants, if given in moderate quantities. This is barley water. The starch it contains is small in amount, and is held in a state of very fine division. When barley water is mixed with milk in equal proportions, it ensures a fine separation of the curd, and is at the same time a harmless addition to the diet. Isinglass or gelatine, in the proportion of a teaspoonful to the bottleful of milk and water, may also be made use of, and will be found to answer the purpose well.

Farinaceous foods, in general, are, as has been said, injurious to young babies, on account of the deficiency during the first months of life of the secretions necessary for the conversion of the starch into dextrine and grape-sugar—a preliminary process which is indispensable to absorption. If, however, we can make such an addition to the food as will insure the necessary chemical change, farinaceous matter ceases to be injurious. It has been found that by adding to it malt in certain proportions the same change is excited in the starch artificially as is produced naturally by the salivary and pancreatic secretions during the process of digestion. The employment of malt for this purpose was first suggested by Mialhe in a paper read before the French Academy in 1845, and the suggestion was put into practice by Liebig fifteen years later.

"Liebig's Food for Infants" contains wheat flour, malt, and a little carbonate of potash, and has gained a well-deserved celebrity as a food for babies during the first few months of life. The best form with which I am acquainted is that made by Mr. Mellin, under the name of "Mellin's Extract for preparing Liebig's Food for Infants." In this preparation, owing to the careful way in which it is manufactured, the whole of the starch is converted into dextrine and grape-sugar, so that the greater part of the work of digestion is performed before the food reaches the stomach of the child. Mixed with equal parts of milk and water, this food is as perfect a substitute for mother's milk as can be procured, and is readily digested by the youngest infants. It very rarely, indeed, happens that it is found to disagree.

In all cases, then, where a child is brought up by hand, milk should enter largely into his diet, and during the first few months of life he should be fed upon it almost entirely. If he can digest plain milk and water, there is no reason for making any other addition than that of a little milk-sugar and cream; but in cases where, as often happens, the heavy curd taxes the gastric powers too severely, the milk may be thickened by an equal proportion of thin barley-water, or by adding to each bottleful of milk and water a teaspoonful of isinglass or of "Mellin's Extract."

Having fixed upon the kind of food which is suitable to the child, we must next be careful that it is not given in too large quantities, or that the meals are not repeated too frequently. If the stomach be kept constantly overloaded, even with a digestible diet, the effect is almost as injurious as if the child were fed upon a less digestible food in more reasonable quantities. A healthy infant passes the greater part of his time asleep, waking at intervals to take nourishment. These intervals must not be allowed to be too short, and it is a great mistake to accustom the child to take food whenever he cries. From three to four ounces of liquid will be a sufficient quantity during the first six weeks of life; and of this only a half or even a third part should consist of milk, according to the child's powers of digestion. After such a meal the infant should sleep quietly for at least two hours. Fretfulness and irritability in a very young baby almost always indicate indigestion and flatulence; and if a child cries and whines uneasily, twisting about his body and jerking his limbs, a fresh meal given instantly, although it may quiet him for the moment, will, after a short time, only increase his discomfort. During the first six weeks or two months, two hours will be a sufficient interval between the meals; afterwards this interval can be lengthened, and at the same time a larger quantity may be given at each time of feeding. No more food should be prepared at once than is required for the particular meal. The position of the child as he takes food should be half reclining, as when he is applied to his mother's breast, and the food should be given from a feeding-bottle. When the contents of the bottle are exhausted, the child should not be allowed to continue sucking at an empty vessel, as by this means air is swallowed, which might afterwards be a source of great discomfort. The feeding apparatus must be kept perfectly clean. The bottle should be washed out after each meal in water containing a little soda in solution, and must then lie in cold water until again wanted. It is desirable to have two bottles, which can be used alternately.

At the age of six months farinaceous food may be given in small quantities with safety, if it be desired to do so; and in some cases the addition of a moderate proportion of wheaten flour to the diet is found to be attended with advantage. The best form in which this can be given is the preparation of wheat known as "Chapman's entire wheaten flour." This is superior for the purpose to the ordinary flour, as it contains the inner husk of the wheat finely ground, and is therefore rich in phosphates and in a peculiar body called cerealin, which has the diastatic property of changing starchy matters into dextrine. This flour should be slowly baked in an oven until it crumbles into a light grayish powder. At first no more than one teaspoonful should be given once or twice a day, rubbed up (not boiled) with milk. If there be much constipation, fine oatmeal may be used instead of the baked flour.

After the eighth month a little thin mutton or chicken broth or veal tea may be given, carefully freed from all grease. After twelve months the child may begin to take light puddings, well-mashed potatoes with gravy, or the lightly boiled yolk of an egg; but no meat should be allowed until the child be at least sixteen months old. Every new article of food should be given cautiously, and in small quantities at first, and any sign of indigestion should be noted and a return be made at once to a simpler method of feeding.

During all this time the child should be kept scrupulously clean, and his nursery should be well ventilated and not be kept too hot. He should be washed twice a day from head to foot, once with soap. The air of his bedroom should be kept sweet and pure during the day, and at night, if the weather does not allow of an open window, a lamp placed in the fender will insure of a sufficient exchange of air. The child should pass as much of his time as possible out of doors, and while every care is taken to guard his sensitive body against *sudden* changes of temperature, he must not be covered up with too heavy clothing and shut off from every breath of air for fear of his catching cold. A child ought to lie cool at night, and the furniture of his cot, although sufficiently thick to insure necessary warmth, should not be cumbersome, so as to be a burden. If the above directions are carefully carried out—and the mother should herself see that they are attended to—few cases will be found to present any difficulty in their management. Exceptional cases, however, are sometimes met with where special sources of embarrassment may arise. These I propose to consider in a future paper.—*Sanitary Record.—The Sanitarian, Jan.*, 1875.

